# Combined Role of Organic Ligands and Ultrasound on the Dissolution of Phlogopite at pH 4 and 7

**DOI:** 10.1021/acs.langmuir.4c04307

**Published:** 2025-06-06

**Authors:** Mahtab Akbarzadeh Khoei, Recep Kurtulus, Mohammad I. M. Alzeer, Juho Antti Sirviö, Juho Yliniemi

**Affiliations:** † Fibre and Particle Engineering Research Unit, 6370 University of Oulu, P.O. Box 4300, 90014 Oulu, Finland; ‡ Faculty of Engineering, Department of Materials Science and Engineering, Afyon Kocatepe University, Afyonkarahisar 03204, Turkey

## Abstract

Phlogopite, a mineral produced in large quantities by the mining industry, has potential applications in the cement industry, fertilizers, and carbon storage, but its use is limited by the slow dissolution caused by its stable crystalline structure. This study investigated the combined effect of ultrasound waves and organic ligands (citrate, oxalate, and ethylenediamine) on the extraction of elements from phlogopite at acidic and neutral pH using batch dissolution experiments. It was hypothesized that sonicated samples would exhibit improved dissolution compared to mechanically stirred samples. The results showed that the dissolution of phlogopite increases in sonicated samples in the presence of an organic ligand. This enhancement depends on ligand type, with the effect being notably higher in the case of the sample containing citrate. In addition, pH plays an important role, as element extraction percentages were significantly higher at an initial pH of 4 compared to pH 7. The surface study suggests that ultrasound waves affect the morphology of phlogopite; however, there is no noticeable effect on preventing the reaccumulation of elements on the surface. The findings suggest that sonication, along with organic ligands, could be a useful processing step for the utilization of phlogopite in different applications. This research provides novel insights into the complex dynamics of enhanced dissolution and the interfacial interactions involving sonication and organic ligands.

## Graphical abstract

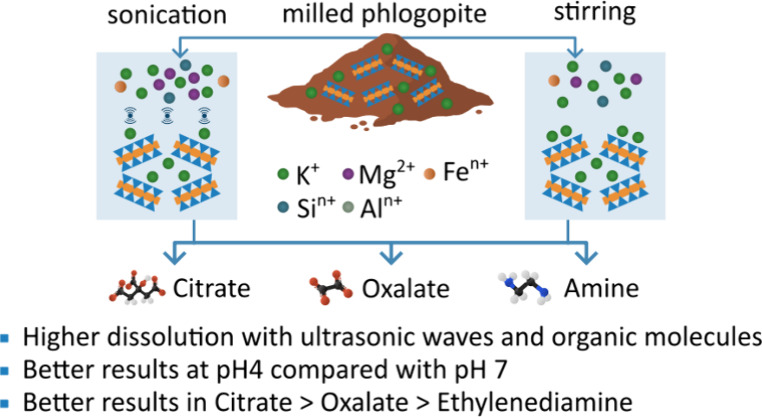

## 1 Introduction

The circular economy promotes the best practices of waste management to decrease the impacts of industrial side stream disposal and the depletion of virgin resources. The key strategy is to utilize such wastes effectively in applications where the volume of generated side streams matches the volume utilized by the application. From this perspective, for aluminosilicate-type side streams, utilization in the cement industry, and carbon capture and storage have potential.^1–4^

A phlogopite-rich side stream, produced during mining activities, is one of the aluminosilicate mineral wastes that lack existing utilization pathways. Based on its chemical composition, i.e., high Si, Al, and Mg content, the potential utilization applications of phlogopite could be the cement industry,^5,6^ K extraction and further utilization as fertilizer,^7,8^ and as a CO_2_ sink.^9–11^ In the cement industry, the released Si and Al from phlogopite can contribute to the formation of calcium silicate hydrate (C–S–H), which is a key component in cement. Additionally, Ca is essential for the overall strength and durability of the cement.^12^ K released from phlogopite can be enriched or precipitated as K_2_SO_4_, which is a valuable fertilizer. This process can enhance the agricultural value of the extracted K.^7,8^ In the mineral carbonation process, cations such as Ca and Mg are crucial as they form stable carbonates (e.g., CaCO_3_ and MgCO_3_). These carbonates are essential for long-term CO_2_ sequestration.^13^ The first step for its utilization is efficient dissolution. Phlogopite is a mica group mineral exhibiting a crystalline structure with sheet-like layers comprising Si– and Al–O tetrahedra and Mg–O octahedra. K ions are sandwiched between these layers.^14^ The crystalline structure of the mineral impedes the penetration of solvents or reactants, thereby preventing effective breakdown. Additionally, its chemical stability and resistance to dissolution present significant challenges.^15,16^

The dissolution of silicate materials is influenced by a multitude of factors, including the composition and structure of the mineral, temperature, solution pH, ionic strength, and composition of the solution.^17–21^ In addition, the mineral dissolution process and extraction of elements can be affected by complexing ligands.^21,22^ The effect of ligands on dissolution is often attributed to their ability to complex with dissolved cations in solution, thus preventing their reaccumulation on the mineral surface. Additionally, ligands can polarize and weaken the bond between the cation and the mineral lattice. Their adsorption on the mineral surface, which can be either physical or chemical, can also play a significant role.^23^ Certain ligands, such as carboxylates, can enhance the solubility of minerals like quartz, suggesting that chelating ligands could complex with Si tetrahedra, and other cations present in the mineral lattice.^19,22,24–26^ In other words, ligands can enhance mineral dissolution rates through three primary mechanisms: (1) metal–ligand (M–L) complex formation in the solution, which involves ligands binding with metal ions, reducing the concentration of free metal ions at the mineral surface and driving further dissolution to restore equilibrium.^27^ (2) Surface complexation occurs when ligands interact directly with metal ions on the mineral surface or with metal ions that have been adsorbed on negatively charged silicate surface, weakening the metal–oxygen bonds and facilitating the release of metal ions into the solution.^26^ (3) Formation of surface complexes that passivate or “protect” the surface from further dissolution. This highlights the multifaceted role of ligands in the dissolution process of minerals.^28^.

In order to increase the surface reactivity of minerals, different treatment methods such as mechanochemical and thermal activation have been used.^6,8,29^ Sonication is regarded as an environmentally friendly alternative technique. It utilizes inaudible ultrasound waves to travel through elastic media such as water and slurry, enhancing mineral dissolution.^30,31^ When a solid–liquid slurry is exposed to ultrasound, the ultrasonic vibrations trigger a physical phenomenon known as “acoustic cavitation”.^11,32,33^ Acoustic cavitation involves the formation of bubble cavities during the rarefaction cycles and their collapse during the compression cycles under the influence of ultrasonic vibrations. The implosion of these bubbles in the acoustic field leads to the creation of microjets. These microjets generate extremely high local temperatures and pressures, of nearly 5000 K and 500 atm respectively, with rapid heating and cooling rates exceeding 1010 K/s.^34^ The microjets cause surface pitting and particle fragmentation when they encounter particles. This facilitates the diffusion of reagents into the particles and accelerates reactions such as dissolution.^34–36^ In addition, ultrasonic waves can effectively remove impurities from mineral surfaces. By inducing cavitation, the shock waves generated by collapsing bubbles exert intense pressure on the impurity layer. This disrupts adsorption between the impurities and the mineral surface, causing the impurity layer to detach. Additionally, stable cavitation scrubs the surface, and microbubbles penetrate mineral cracks, dislodging the impurities.^37^

Moreover, the composition of the mineral and its crystallinity dictate how acoustic stimulation frequency can affect the structure and the dissolution phenomenon. In the studies conducted under constant temperature and agitation in ultrasonic conditions, several minerals were examined-including calcite, dolomite, obsidian, albite, and quartz. The findings revealed that the energy required to break the Si–O bond (799.6 ± 13.4 kJ/mol) was significantly higher than for the Ca–O (383.3 ± 5.0 kJ/mol) or Mg–O bonds (358.2 ± 7.2 kJ/mol).^11,31^ Existing literature indicates that ultrasonic treatment can enhance particle reactivity by increasing particle collisions, thereby improving dissolution processes. This effect has been observed in various applications, such as the leaching of gold from ores and the dissolution of minerals such as quartz and calcite.^38,39^ Hence, the dissolution properties of minerals can be improved, depending on their crystal structure. To conclude, both organic ligands and sonication can affect the mineral surface and mineral dissolution in different ways. Here, we hypothesize that, if these two effects are combined, it further increases the dissolution of the mineral—the effect being dependent on the type of ligand, solution pH, and surface chemistry of the mineral.

In this research, we carried out batch dissolution experiments at pH 4 and pH 7 and examined the impact of citrate, oxalate, and ethylenediamine, as well as the effect of sonication, on the dissolution and surface chemistry of phlogopite. The incorporation of ultrasound as an auxiliary technique in this study provides a novel approach to understanding the intricate interplay between the physical agitation caused by mechanical energy and ligand-driven dissolution processes. This research provides new insights for understanding the complex dynamics of enhanced dissolution and the interfacial interactions affecting the mineral surface.

## 2 Methods and Materials

### 2.1 Materials

An iron-bearing phlogopite (KMg_2.5_Fe_0.5_(AlSi_3_O_10_)­(OH)_2_) from LKAB, Siilinjärvi (Finland) was used in this study. Sodium oxalate (Merck, Germany), trisodium citrate (Merck, Germany), anhydrous ethylenediamine (C_2_H_8_N_2_) purchased from TCI (Tokyo), and nitric acid (69 wt %) for analysis obtained from Merck (Darmstadt, Germany) were used. The phlogopite was milled using a Retsch ball mill PM 200 to increase its surface area, reduce its particle size, and change its mineralogy. The X-ray diffraction and transmission electron microscopy (TEM) results reported in our previous study^40^ showed that milled phlogopite is composed of nanocrystals in an amorphous matrix. The chemical composition as measured by X-ray fluorescence (XRF), the mean particle size distribution (*d*_50_), and the surface area (*S*_BET_) data of the phlogopite used are shown in Table 1.

**Table 1:** Chemical Composition (Analyzed by XRF), Mean Particle Size (*d*_50_), and Surface Area (*S*_BET_) of Phlogopite^40^

oxide	SiO_2_	MgO	Al_2_O_3_	K_2_O	Fe_2_O_3_	CaO	TiO_2_	Na_2_O	P_2_O_3_	sum	d_50_ (μm)	*S*_BET_(m^2^/g)
wt %	39.7	23.1	9.5	8.3	8.7	1.3	0.2	0.1	0.4	97.5	29.23	3.37

### 2.2 Methods

Batch dissolution experiments were conducted with two experimental setups: mechanical stirring using a magnetic stirrer, and sonication where an ultrasonicator was used (details in the following section). All reference and sample batches were prepared at the initial pHs of 4 and 7. To study the effect of ligands on dissolution at these initial pHs, two types of solutions were prepared for each pH, one without any ligand as reference and the other one including the ligand as sample batches. The sample solutions were prepared by adding the organic ligand and a suitable amount of 0.1 M nitric acid to reach the desired initial pH. The total volume of the solution for all the samples was the same to obtain a constant liquid-to-solid ratio of 400, resulting in the initial solid surface area-to-solution volume ratio of 14070 m^–1^ (0.25 g of phlogopite in 100 mL of Milli-Q water). In the sample batches, the concentration of the ligand at each pH was as shown in Table 2; the difference in the values is due to keeping a liquid-to-solid ratio of 400 for all the samples.

**Table 2:** Sample Codes, Ligand Concentration, and Initial and Final pH after the Experiment^a^

batch code	initial pH	final pH	ligand (M)
untreated phlogopite	N.A.	N.A.	N.A.
R-NL4	4.0	5.6	N.A.
S-NL4	4.0	5.7	N.A.
R-NL7	7.0	7.3	N.A.
S-NL7	7.0	7.4	N.A.
R-Ox4	4.0	5.2	0.06
S-Ox4	4.0	6.8	0.06
R-Ox7	7.0	7.8	0.08
S-Ox7	7.0	8.7	0.08
R-Cit4	4.0	5.3	0.04
S-Cit4	4.0	5.6	0.04
R-Cit7	7.0	7.4	0.08
S-Cit7	7.0	7.6	0.08
R-En4	4.0	5.4	0.02
S-En4	4.0	5.8	0.02
R-En7	7.0	7.5	0.03
S-En7	7.0	7.8	0.03

a The accuracy of pH measurements is ± 0.1. The sample codes are abbreviated according to their initial pH (4 and 7), mixing method (stirrer = R or sonicated = S), and ligand used (NL = no ligand, Ox = oxalate, Cit = citrate, En = ethylenediamine).

An additional set of dissolution experiments was conducted to compare the individual effects of sonication, final pH, ligand type and concentration on mineral dissolution and element extraction (Table 3). Experiments R2-Ox4, S2-Ox4, R2-En4, and S2-En4 were conducted with a ligand concentration of 0.04 M, similar to R-Cit4 and S-Cit4. Experiments R2-En7 and S2-En7 were performed with a ligand concentration of 0.08 M, comparable to the former Ox7 and Cit7 samples. The experiment S2-Ox7 was specifically designed to control the final pH. Therefore, 0.1 M nitric acid was added to the sodium oxalate samples, while 1 M nitric acid was used to maintain the intended concentration of ethylenediamine at pH 4 and 7. The use of 0.1 M nitric acid for the ethylenediamine samples was not feasible, as the resulting volume change would affect the Reynolds number and compromise the validity of the experiments. All samples had an initial solid surface area-to-solution volume ratio of 14070 m^–1^. To achieve similar final pH values, nitric acid was added to S2-Ox7 sample every 10 min during sonication. This process involved intermittently stopping and restarting the sonication, with nitric acid added to control the pH.

**Table 3:** Sample Codes, Ligand Concentration, and Initial and Final pH of Additional Experiments^a^

batch code	initial pH	final pH	ligand (M)
R2-Ox4	4.0	5.1	0.04
S2-Ox4	4.0	5.2	0.04
S2-Ox7	7.0	7.6	0.08
R2-En4	4.0	5.1	0.04
S2-En4	4.0	5.3	0.04
R2-En7	7.0	7.3	0.08
S2-En7	7.0	7.4	0.08

a The accuracy of pH measurements is ± 0.1. The sample codes are abbreviated according to their initial pH (4 or 7), mixing method (stirrer = R2 or sonicated = S2), and the ligand used (NL = no ligand, Ox = oxalate, and En = ethylenediamine).

#### 2.2.1 Stirred Systems

After preparing the mixture batches, the R-sample batches were stirred for 1 h using a 0.5 cm stir bar at 330 rpm.

#### 2.2.2 Sonicated Systems

To study the effect of ultrasound waves on the dissolution and surface characterization of the mineral, the S-samples were sonicated for 1 h with a Hielscher UP 400s ultrasonicator (Germany) using a 7 mm titanium tip, at 24 kHz frequency, 60% amplitude, and 0.5 cycle.

The Reynolds number (*Re*) is a dimensionless quantity in fluid mechanics that helps predict fluid flow patterns in different situations by measuring the ratio between inertial and viscous forces. It is used to predict the transition from laminar to turbulent flow and is used in the scaling of similar but different-sized flow situations. The Reynolds number of stirred and sonicated samples can be calculated using the following eqs 1 and 2, respectively^11,41,42^ :

$$Re=\frac{V_{R}.D.\rho }{v}$$ 1

$$Re=\frac{V_{S}.L.\rho }{v}$$ 2

where: *Re* is the Reynolds number, ρ is the density of the fluid (kg/m^3^), *V*_*R*_ is the average circulation velocity (m/s), *D* is the stirrer bar diameter (m), ν is the kinematic viscosity of the fluid (kg/m s), *V*_*S*_ is the average circulation velocity (m/s), *L* is the horn tip diameter (m).

eq 3 was used to calculate *V*_*S*_, where *T* is the beaker diameter (m), *Z* is the liquid height (m), and θ_min_ is the average mixing time (s).^42^

$$V_{S}=\frac{5T+10Z}{\theta_{\min}}$$ 3

In order to have equivalent conditions of convection and an equal Reynolds number in all of the sample batches, a Reynolds number of around 3900 was maintained to have a normal solution flow.^34,41,42^ It should be noted that the Reynolds number and ligand concentrations may not be optimal. However, we tried to use the most relevant conditions possible and maintained a fixed Reynolds number and initial pHs across all samples to ensure consistency.

Following the dissolution experiments, the suspensions were filtered with a 0.2 μm polyether sulfone filter. After filtration, no rinsing was performed on the solid residue retained on the filter. This approach was taken to ensure that all solution species present during the experimental conditions were retained on the sample surface. The resulting filtrates were acidified to achieve a pH below 2 by gradually adding HNO_3_ (2% wt/wt) with a pipet. The concentrations of Si, Al, Fe, Mg, and K in the filtrates were then analyzed using ICP-OES in accordance with EN ISO 11885 standards and the replicated tests demonstrated a ≤ ± 5% variation in the data.

eq 4 was used to calculate the extraction percentage^20^:

$$\mathrm{Extraction}\;\mathrm{percentage}\;\mathrm{calculation}\;\mathrm{of}\;\mathrm{element}\;i=C_{i}\times V\times \frac{1}{X_{i}\times m_{\mathrm{sample}}}\times 100$$ 4

Here, *C*_*i*_ represents the concentration of the element in the solution determined through ICP-OES analysis (g/L), *V* denotes the volume of the solution (L), *m*_sample_ signifies the mass of the raw material (*g*), and *X*_*i*_ represents the mass fraction of element *i* in the raw material based on XRF analysis (−).

#### 2.2.3 Analytical Techniques

The solids obtained after filtration were dried overnight at 90 °C and stored in a desiccator until analysis by X-ray photoelectron spectroscopy (XPS). XPS data were acquired utilizing the Thermo Fisher Scientific ESCALAB 250Xi XPS system, equipped with a monochromatic Al Kα X-ray source with an energy of 1486.68 eV. Scans were conducted with a pass energy of 150 eV and a step size of 0.5 or 1.0 eV. Analysis of the spectra was performed using Avantage software v.5.976, with calibration achieved by assigning the characteristic adventitious carbon C 1s peak energy to 284.88 eV.

Zeta potential measurements of the samples were conducted both before and 10–30 min after concluding the dissolution experiments, employing the Zetasizer Pro Blue Label (Malvern Panalytical, UK) and ZS Xplorer software v.1.3.2.27. After the experiment, 1 mL of the sample mixture was analyzed using a DTS 1070 disposable folded capillary cell. The zeta potential, measured three times at a consistent temperature of 25 °C, involved a 2 min stabilization period before each measurement, with a 1 min gap between measurements. Automated optimized parameters and a versatile analysis model were utilized. The zeta potential values, computed using Smoluchowski’s theory modified for electrophoretic light scattering, were averaged from three consecutive measurements, taking approximately 10 min in total.

Dried powder samples were analyzed using diffuse reflectance infrared Fourier transform (DRIFT) spectroscopy. The analysis used a spectral range from 500 to 4000 cm^–1^, which is a typical range for many organic and inorganic materials. The Bruker Vertex v80, a high-quality infrared spectrometer with high reliability and accuracy, was the instrument selected for this analysis. In total, 60 scans were performed for each sample and each spectral scan was conducted with a resolution of 8 cm^–1^.

Transmission electron microscopy (TEM) was done using a JEOL JEM-2200FS transmission electron microscope, equipped with an energy-dispersive X-ray spectrometer. The microscope operated at an acceleration voltage of 200 kV and had a functional depth range of 1–2 μm.

## 3 Results

Figure 1a presents the dissolution results of all the samples at pH 4. All the ligands improved the extraction percentage, and combined use of sonication and ligand improved the extraction even further. The extraction percentage of the samples including the ligands was citrate > oxalate > ethylenediamine with some variation for different elements. It is noteworthy that the ranking does not take into account the variation of ligand concentration which affects the dissolution. Given the constraints of the experimental setup, it was focused on maintaining the same initial pH and Reynolds number to ensure the validity of the results. Combined use of citrate and sonication approximately doubled the extraction percentage of all elements at pH 4, showing their high synergistic impact. The presence of ethylenediamine increased the extraction of all elements, but the greatest effect was observed for Mg and K with further improved extraction with sonication, which shows that the combined use of ligand and sonication also exhibited certain selectivity in the extraction.

**Figure 1:**
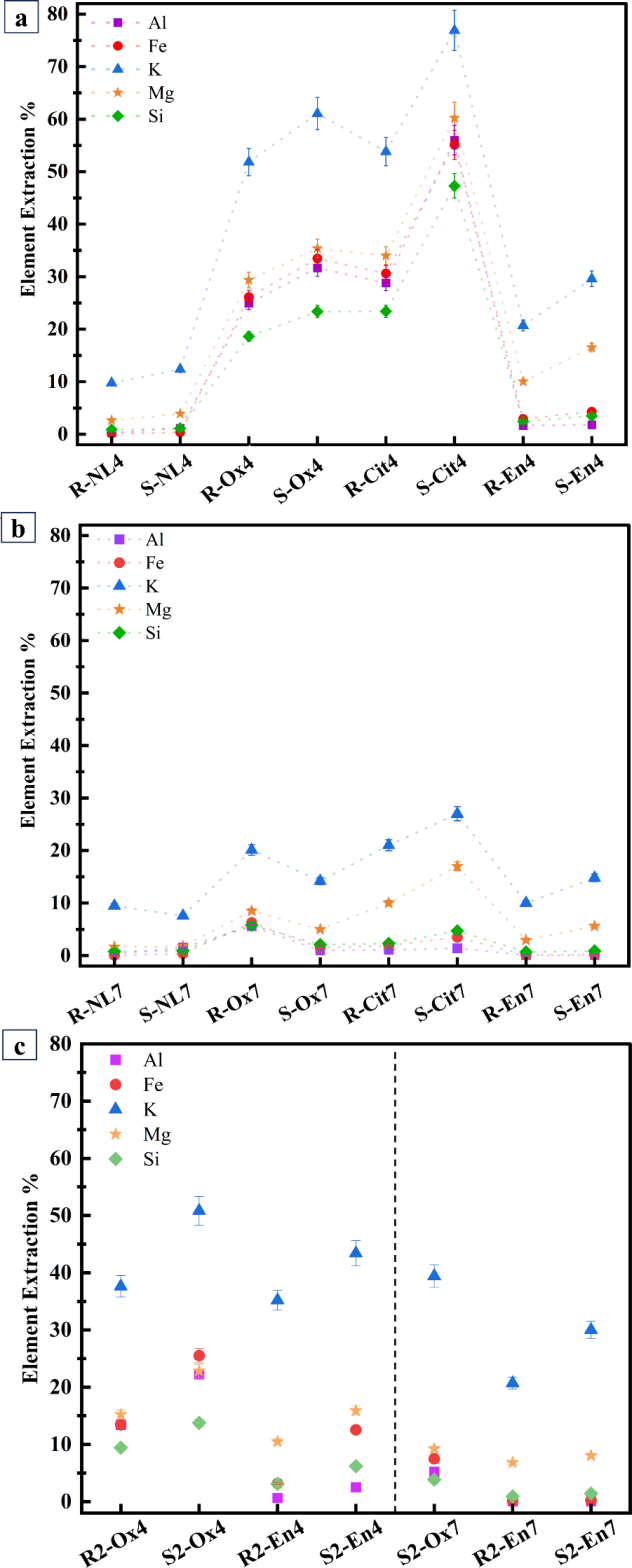
Batch dissolution results of samples (a) at initial pH 4, (b) at initial pH 7, and (c) for additional experiments as element extraction % as calculated by eq 4. The sample codes are abbreviated according to their initial pH (4 or 7), mixing method (stirrer = R or sonicated = S), and the ligand used (NL = no ligand, Ox = oxalate, Cit = citrate, En = ethylenediamine).

Extraction of elements was lower at pH 7 than at pH 4 (Figure 1b). Ligands increased the extraction of elements at pH 7 as well, but the effect was less pronounced compared to pH 4. The combined use of citrate or ethylenediamine with sonication increased the extraction at pH 7, whereas the combination of oxalate and sonication did not. This demonstrates that the synergistic effect of ligand and sonication is dependent on the pH of the solution. According to Table 2, despite the higher citrate concentration at pH 7, dissolution was not as significant as that observed with a lower citrate concentration at pH 4. This suggests that the ligand dosage is sufficient in both samples and not a limiting factor in the dissolution mechanism.

K as an interlayer element had the highest extraction percentage in all the sample batches. The highest extraction percentages for K were achieved in sonicated samples at pH 4 where about 75% citrate and 60% oxalate were added. Comparing these results with the stirred samples, the contribution of sonication resulted in an increase of K dissolution of up to 1.5x and 1.2x. In sonicated samples with ethylenediamine at pH 4, nearly 32% of K was extracted, which is about 1.4 times more than in the stirred samples. Likewise, K extraction increased in sonicated samples at pH 7 when organic ligands were present, compared to those that were stirred.

Comparing Figure 1c with 1a, it is evident that ligands containing carboxylate groups enhance dissolution more effectively than ethylenediamine with amine groups. Sonication remarkably contributes to dissolution, particularly for K. As shown in Figure 1c, when comparing the dissolution yields of pH 4 and pH 7 samples, it is evident that dissolution is higher in pH 4 samples. Ligand concentration impacts dissolution yield as well; specifically, in the cases of R2-Ox4 and S2-Ox4, lower ligand concentrations result in reduced dissolution yields compared to our former results (Figure 1a). However, for the En4 and En7 samples, the higher acid consumption makes it challenging to determine whether the increased dissolution yield is solely due to the ligand concentration. Furthermore, the presence of nitrate ions may also contribute to the dissolution process. In S2-Ox7, the increase in K extraction compared to Figure 1b is attributed to the addition of nitric acid during sonication to control the final pH.

The zeta potential values of phlogopite before and after the dissolution experiments are presented in Figure 2. In general, zeta potential is influenced by (1) the type and number of surface groups such as >Si–O– and >Al–O­(H), where ‘>’ indicates the surface; (2) solution composition, i.e., pH and concentration of ions and ligands and their charge; and (3) interactions between ions and ligands and the surface groups. In samples containing trisodium citrate or sodium oxalate with an initial pH of 4, the concentration of Na^+^ ions and their interaction with the surface of phlogopite results in a slightly less negative initial zeta potential compared to the R-NL4 and S-NL4 samples. In the R-En4 and S-En4 samples, the speciation and protonation of ethylenediamine cause a near neutral initial zeta potential.

**Figure 2:**
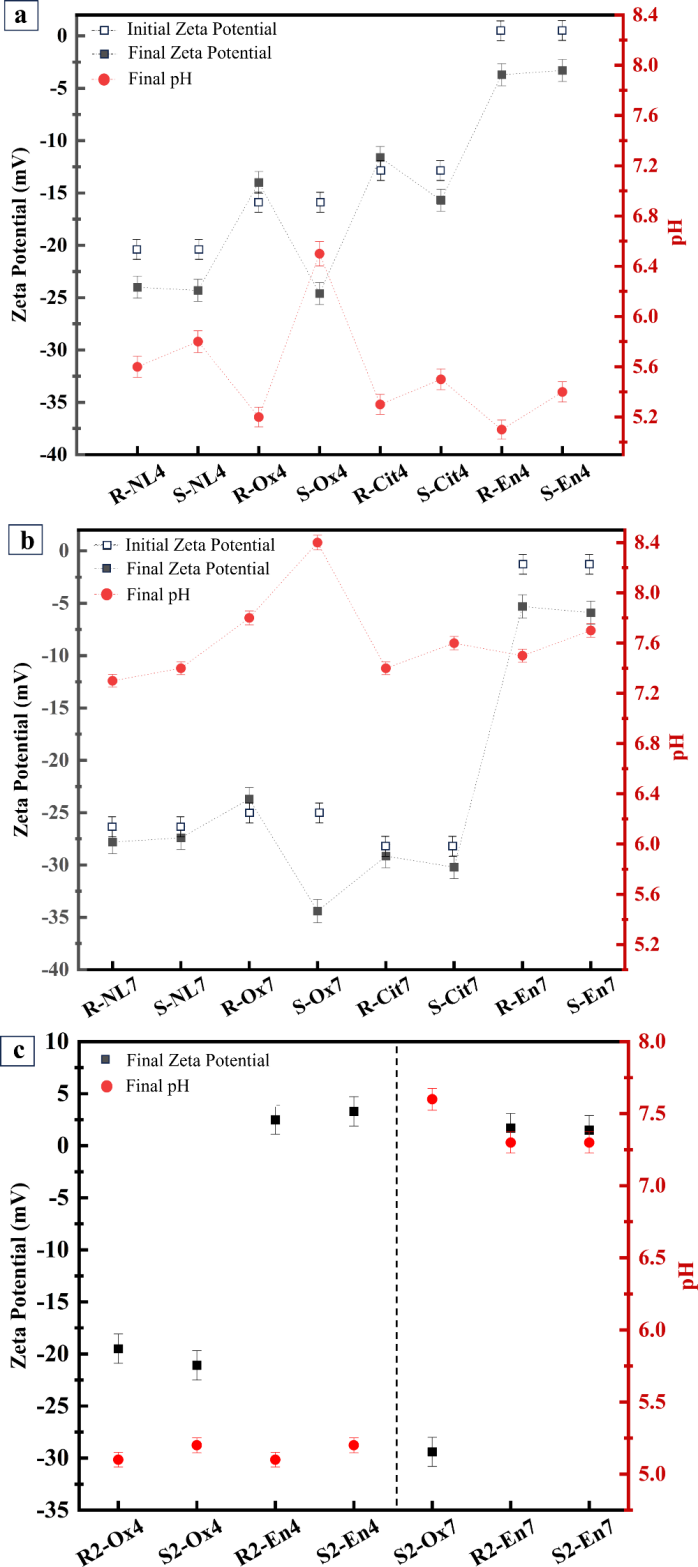
Initial zeta potential (white squares), final zeta potential (black squares) and final pH (circles) values of samples at (a) initial pH 4 and (b) initial pH 7. Final zeta potential and final pH values of (c) additional experiments. The sample codes are abbreviated according to their initial pH (4 or 7), mixing method (stirrer = R or sonicated = S), and the ligand used (NL = no ligand, Ox = oxalate, Cit = citrate, En = ethylenediamine).

Figure 2a shows that sonication of samples with an initial pH of 4 resulted in a lower final zeta potential and a higher final pH. Zeta potential was also influenced by the presence and type of ligand. The zeta potential of the sonicated samples with respect to the ligand used shows that in the S-Ox4 system, the factors resulted in a more negative zeta potential value, whereas in the S-En4 system, the zeta potential is near neutral.

A comparison of Figure 2a,b reveals that the zeta potential is more negative as the final pH rises to the range of 7.2–8.4. This variation in pH, along with the speciation of the ligands in the solution interacting with the surface affects the measured zeta potential. The primary aim of presenting these data is to gain insights into the possible interactions between the ligands and the surface of phlogopite or the cations adsorbed on the surface. Considering the initial and final zeta potential values, the dissolution of cations and changes in their concentration in the solution do not significantly affect the zeta potential. However, the speciation of ligands at different pH levels and their interaction with the cations near the surface influence the zeta potential. Similarly, Figure 2c demonstrates that the zeta potential of the additional experiments with similar ligand concentrations after dissolution is primarily influenced by the final pH and ligand speciation. That is, in En-4 and En-7 samples prepared using higher acid concentration, there is a higher concentration of protonated ethylenediamine in the system affecting the zeta potential. Likewise, in R2-Ox4, S2-Ox4, and S2-Ox7 samples, the final pH and oxalate speciation are the determining factors. However, sonication does not appear to influence the zeta potential of the samples.

The surface elemental ratios of the solid surface determined by XPS analysis, presented in Figure 3, show that, in general, the sonicated samples have lower metal/Si ratios compared to the stirred samples. However, despite the high extraction percentages in certain samples, for example, with citrate and sonication at pH 4, the high extraction is not reflected drastically in the metal/Si ratios as determined by XPS. This is probably because XPS can detect elements only in the first few nanometers below the surface, whereas the extracted elements lie much deeper beneath the surface. In our previous work,^40^ we estimated the depth where K is extracted to be ∼17 μm at pH 4.

**Figure 3:**
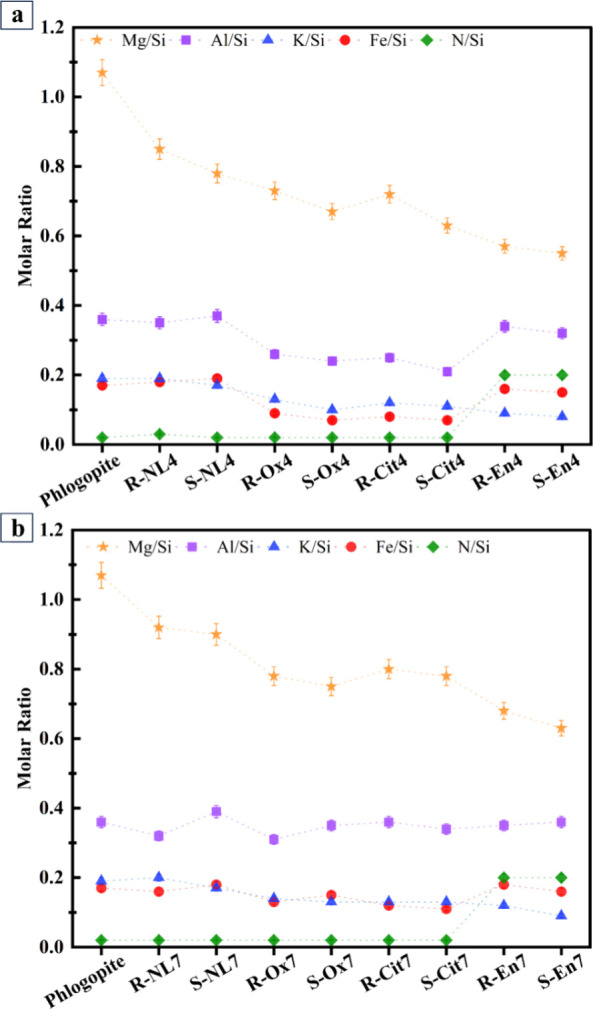
XPS molar ratio results of samples and untreated phlogopite (Phlogopite) with a) initial pH 4 and b) initial pH 7. The sample codes are abbreviated according to their initial pH (4 or 7), activation method (stirrer = R or sonicated = S), and the ligand used (NL = no ligand, Ox = oxalate, Cit = citrate, En = ethylenediamine).

As Figure 3a indicates, the Mg/Si ratio showed the most noticeable changes and was in line with the extraction percentage shown in Figure 1a. K/Si showed a similar trend to Mg/Si; however, the changes were less noticeable. Al/Si and Fe/Si showed comparable patterns, i.e., the metal/Si decreased in the samples containing oxalate and citrate but increased in the presence of ethylenediamine.

When the initial pH was 7, the metal/Si ratios increased slightly, which is in line with the extraction percentage of elements at this pH. Mg/Si shows the highest variations, and this ratio decreases in the presence of ligands. In the case of Al/Si and Fe/Si, the changes are similar and less noticeable than with the pH 4 samples. Like K/Si in the pH 4 samples, the variations of K/Si between pH 7 samples are small and the ratios are close to each other.

In addition, it is noteworthy that the N/Si ratio in the samples containing ethylenediamine indicates that, in both stirred and sonicated samples, the ligand is present on the surface and the activation method utilized did not affect this phenomenon.

The FTIR spectra of all the batches are presented in Figure 4. The spectra of oxalate samples in Figure 4a show that a sharp peak appears at 1310 cm^–1^, corresponding to the symmetric stretching of C–O bonding.^37^ The peaks at 1435 and 1645 cm^–1^ are related to carbonated species^45^ and the O–H bending of water, respectively.^46,47^ The peaks at 1571 and 1610 cm^–1^ in citrate and oxalate samples, respectively, are related to the C=O asymmetric stretching vibration, suggesting that oxalate and citrate may be present on the surface of the solid residue.^43,44,48–50^

**Figure 4:**
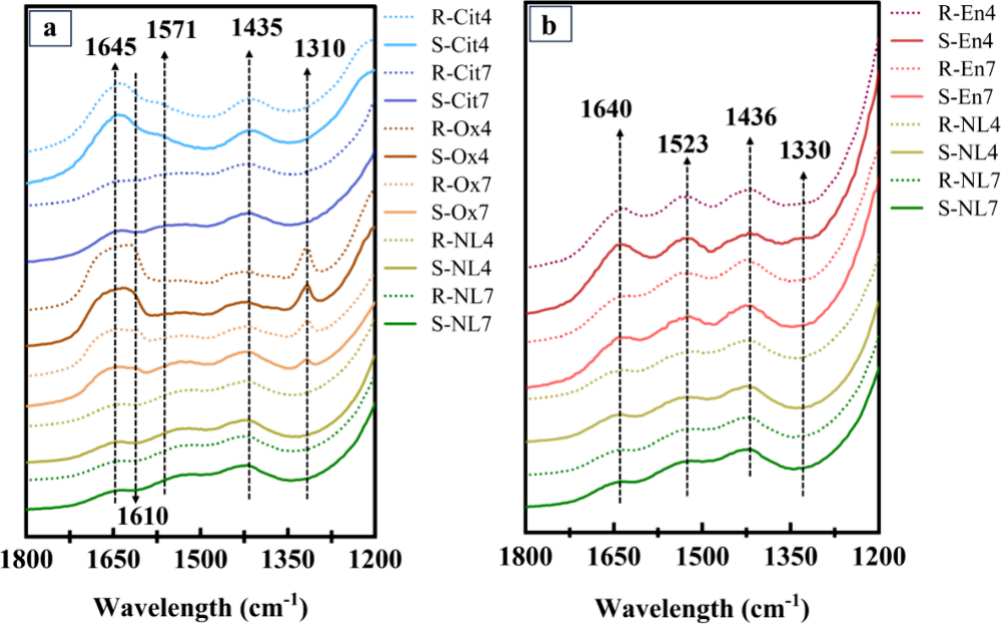
FTIR spectra of (a) reference, oxalate and citrate samples, and (b) reference and ethylenediamine samples. The sample codes are abbreviated according to their initial pH (4 or 7), mixing method (stirrer = R or sonicated = S), and the ligand used (NL = no ligand, Ox = oxalate, Cit = citrate, En = ethylenediamine).

Figure 4b shows the spectra of samples containing ethylenediamine and the reference samples. Although there seems to be no significant changes in the spectra and peaks, one broad peak can be observed at 1330 cm^–1^ which may be related to C–H bonding in ethylenediamine,^51^ indicating its presence on the surface of the phlogopite. The peak at 1436 cm^–1^ is attributed to the formation of carbonated species.^45^ The broad peak at 1523 cm^–1^ is related to the bending mode of N–H in the amine.^52^ The peak at 1640 cm^–1^ is associated with the O–H bending of the adsorbed water.^46,47^ The full-range (770–3650 cm^–1^) overlapped version of the FTIR spectra is shown in Figure S1.

The XPS C 1s spectra of the samples are shown in Figure 5. The peaks appearing at 284.8 eV are related to C–C bonding, which is associated with the carbon contamination on samples that forms during air exposure, known as “adventitious carbon”.^53,54^ However, the peaks around 288.7 eV may be related to the O–C=O bond in citrate and oxalate, indicating the presence of these ligands on the surface of the phlogopite. The results indicate that, regardless of the mixing method, citrate and oxalate were present on the phlogopite surface after the dissolution experiments.

**Figure 5:**
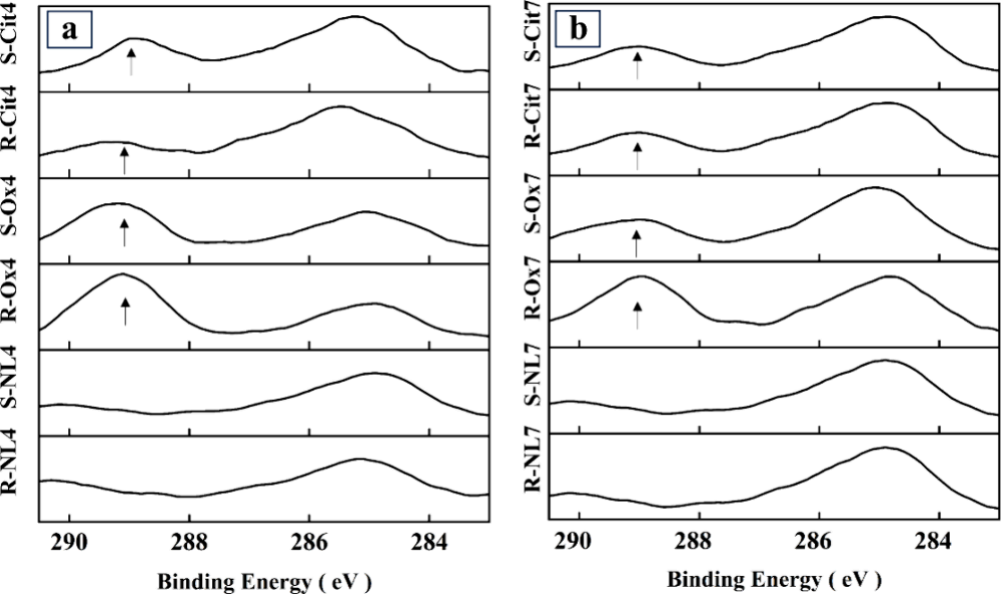
XPS C 1s spectra of reference, oxalate and citrate samples with (a) initial pH 4 and (b) initial pH 7. The sample codes are abbreviated according to their initial pH (4 or 7), mixing method (stirrer = R or sonicated = S), and the ligand used (NL = no ligand, Ox = oxalate, Cit = citrate).

The presence of ethylenediamine on the phlogopite surface is demonstrated by the N 1s and C 1s XPS spectra (Figure 6). Figure 6a shows two peaks at 399.3 and 401.7 eV originating from the NH_2_ and NH_3_^+^ groups of ethylenediamine^40,55^ showing clear evidence that ethylenediamine is present on the phlogopite surface. Additionally, apart from the peak at 284.8 eV, the extra peak at 286.6 eV in the C 1s spectra of ethylenediamine samples representing C–C–N bonding supports the previous claim.^55^ As with the oxalate and citrate samples, the stirring method did not affect the presence of ethylenediamine on the phlogopite surface.

**Figure 6:**
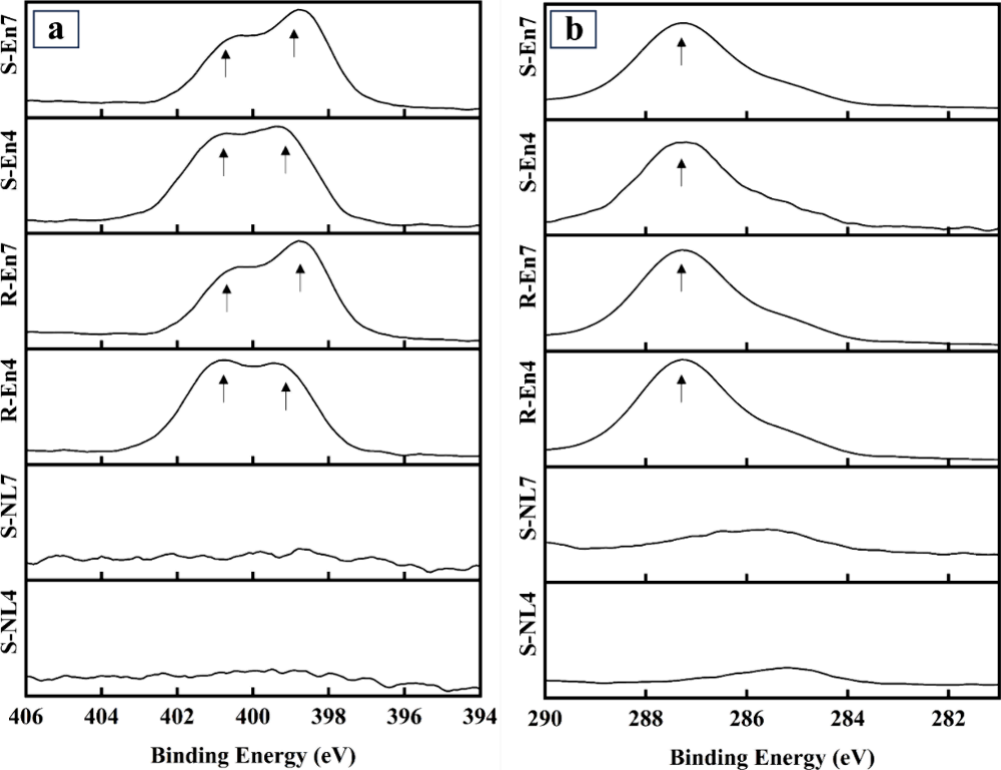
XPS (a) N 1s and (b) C 1s spectra of reference and ethylenediamine samples. The sample codes are abbreviated according to their initial pH (4 or 7), mixing method (stirrer = R or sonicated = S), and the ligand used (NL = no ligand, En = ethylenediamine).

Comparing the TEM images of untreated, stirred, and sonicated phlogopite samples at initial pH 4 (Figures 7 and S2), it is evident that the surface morphology of phlogopite changes after dissolution and upon exposure to ultrasound waves. The longer and thicker layers of nanocrystals are broken down into smaller, thinner ones by sonication. These morphological changes are expected to enhance the extraction percentage by increasing the available surface area. At the same time, there are no clear observable changes in the morphology due to the presence of ligands.

**Figure 7:**
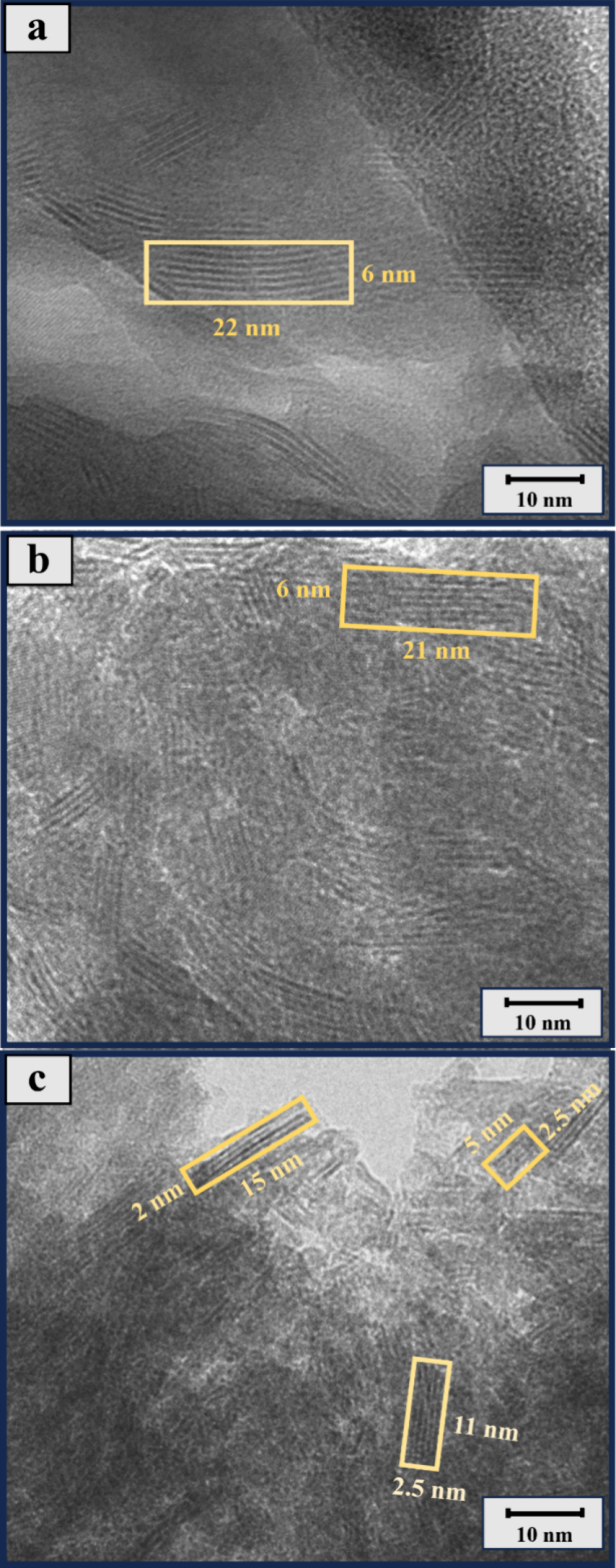
Morphological investigation of samples: (a) milled phlogopite, (b) R-Cit4, and (c) S-Cit4. The sample codes are abbreviated according to their initial pH (4), mixing method (stirrer = R or sonicated = S), and the ligand used (Cit = citrate).

## 4 Discussion

As shown in Figure 8, due to the layered structure of phlogopite and the arrangement of K as an interlayer element,^56^ K has the highest extraction rate. K and other cations are released via proton-metal exchange reactions where protons (H^+^) from the surrounding solution replace the metal cations in the mineral lattice.^57,58^ In a more acidic environment, the concentration of H^+^ is higher, and thus the extraction percentage is higher at pH 4 than at pH 7. The consumption of protons due to proton-metal exchange reactions can be estimated based on the final pH of the solutions. The extraction of cations from the phlogopite framework destabilizes its structure, resulting in the release of Si, as observed in other studies.^59,60^ However, there is a possibility that Si could precipitate back onto the surface and form alteration or passivation layers. When silica is released into the solution, it can reprecipitate onto the mineral surface, forming a Si-rich layer. This layer can act as a passivation layer, potentially slowing down further dissolution by creating a barrier between the mineral surface and the solution.^61,62^

**Figure 8:**
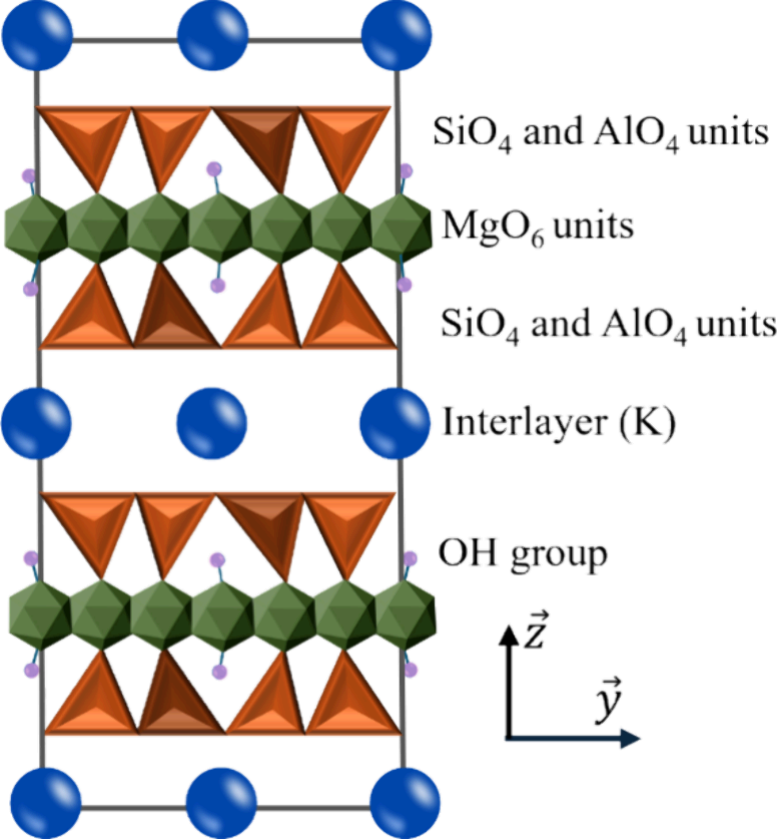
Schematic image of the crystal structure of phlogopite shows the SiO_4_ and AlO_4_ units as orange tetrahedrons and the MgO_6_ units as green octahedrons. The H atoms are depicted as purple spheres, while the K atoms are represented as large blue spheres.

The inclusion of organic ligands enhances dissolution, and this effect is more pronounced with carboxylate ligands than with ethylenediamine. This can be explained by the ability of carboxylate ligands to form complexes with metal ions. The carboxylate group (−COO−) has a strong affinity for metal ions, leading to the formation of soluble metal-carboxylate complexes.^18,21,26^ The high extraction percentages of K and Mg are probably a result of these elements forming complexes with the carboxylate groups in the solution. Al, Fe, and Si are the least extracted elements, which could be explained by the strong Al–O and Si–O bonds of phlogopite that contain Fe in the octahedral holes, linked tightly altogether.^14^ Indirect evidence on complexation can be gathered by comparing the metal/Si ratios of the surface (Figure 3). Samples with oxalate or citrate show lower Al/Si, Fe/Si, Mg/Si, and K/Si ratios. If cations accumulate on Si-rich surfaces, they stabilize them and inhibit dissolution—especially Al, which is known to hinder silicate dissolution.^19^ The lower metal/Si ratios found in the treated samples compared to untreated phlogopite imply that elements were released into the solution, which is consistent with the dissolution outcomes shown in Figure 1a,b. Increased metal/Si ratios may indicate surface precipitation or adsorption, which prevents further dissolution. Ethylenediamine with two amine functional groups does not have such a significant impact on the dissolution of phlogopite, mainly increasing the dissolution of K and Mg. The order of citrate > oxalate > ethylenediamine is logical, considering the fact that citrate as a tridentate chelating ligand contains three carboxylate groups and oxalate has two chelating carboxylate dentates.^18,25^

Furthermore, ultrasound waves promote the formation of microbubbles and cavitation in the solution, which can increase the surface area available for dissolution, thus increasing the concentration of ions in the solution.^35,37^ This was observed by TEM analysis where the sonicated samples showed smaller and thinner nanocrystals compared to the stirred samples. In addition, dissolution is enhanced by the synergistic effect of ultrasound waves with organic ligands, which may be related to the complexation effect of the ligands that contributes to the formation of complexes in the solution. This observation aligns with the findings of Sun et al., who demonstrated that carboxylate ligands enhance mineral dissolution kinetics through strong interactions between dissolved cations and the carbonyl oxygen of the carboxylate radical, as shown by DFT calculation.^63^ Additionally, another study investigated the complexation of citrate with Mg and Fe at different pH levels, indicating that these species begin to form in the solution at pH values greater than 4.^21^ Ultrasound waves can facilitate this process by increasing the mixing and interaction between the ligands and metal ions in solution.^64,65^ When the initial pH was 7, dissolution decreased significantly compared to the samples with initial pH 4; however, it is important to note that the ligands, specifically carboxylates, still contributed to the dissolution process even at neutral pH. Additionally, precipitation of Mg-oxalate, for example, may occur, which could explain the lower ion concentration in the solution.

There was strong experimental evidence based on FTIR and XPS analyses that the ligands interacted and were present on the phlogopite surface. Sonication plays a crucial role in this process by significantly enhancing the mass transport of species within the liquid medium. This enhancement is achieved through the creation of microstreaming and fluid flow, which are induced by the ultrasonic waves. The microstreaming effect generates localized high-velocity liquid jets, improving the overall mixing and distribution of the species in the solution.^34,36,66^ Therefore, sonication facilitates a more efficient transport of ligands to the phlogopite surface, ensuring that they reach and interact with the surface more effectively. The interaction of ligands with the surface is also supported by the zeta potential measurements where ligands increased or decreased the zeta potential depending on the type of ligand, solution pH, and composition. According to the speciation of oxalate and citrate at different pH values, in samples where pH < 6, hydrogen oxalate (HC_2_O_4_^–^) is expected to be the main species in solution. When the pH rises, the main species is C_2_O_4_^2–^.^67^ Similarly, for citrate, in samples where pH < 7, dihydrogen citrate (H_2_C_6_H_5_O_7_^–^) is expected to be the main species in solution. As the pH increases, citrate transitions to hydrogen citrate (HC_6_H_5_O_7_^2–^) and eventually to its fully deprotonated form (C_6_H_5_O_7_^3–^).^68^

In addition, hydrogen citrate could form outer-sphere complexes with the surface sites of phlogopite.^50^ This might lead to a different surface charge distribution compared to the oxalate species, resulting in a lower zeta potential. Therefore, the interaction of these species with the surface affects the zeta potential of the samples. At the same time, the solution composition, particularly the concentration of cations, can interact with the surface. This interaction affects the electrolyte composition during zeta potential measurements, thereby influencing the zeta potential of the phlogopite surface. In samples containing ethylenediamine, in the pH range of 5–11 ethylenediamine is in its monoprotonated form, carrying a + 1 positive charge,^69^ which explains the near-zero zeta potential as it interacts with the surface. This observation aligns with previous research findings on the interaction of monoprotonated ethylenediamine with the surface of phlogopite.^40^ The strong interaction between surface groups and ethylenediamine at the surface might hinder the further progression of dissolution. Considering the negative charge of the silicate surface under these experimental conditions, and the negative charges of citrate and oxalate, it is expected that oxalate and citrate will be repelled from the surface. However, the adsorption of ligands onto the phlogopite surface can occur due to complex interactions between the ligands and the adsorbed cations such as Na^+^ from trisodium citrate or sodium oxalate, or leached cations during dissolution like K^+^, Mg^2+^ and Fe^2+/3+^. These cations screen surface charges by neutralizing the negative charges on the phlogopite surface, reducing the overall surface potential.^70^ This charge screening effect can facilitate the adsorption of ligands by decreasing electrostatic repulsion between the negatively charged surface and the ligands.^72^ Additionally, the cations can interact with surface functional groups such as >Si–O^–^ and >Al–O­(H), facilitating ligand adsorption by acting as bridging entities. This is consistent with DFT studies showing that the carbonyl oxygen of ligand complexes with cations on the mineral surface.^63^ Another possible mechanism is the complexation of ligands with the dissolved cations in the solution. This would lower the free cation concentration in solution, drive dissolution of cations, and prevent the accumulation of cations on the surface of phlogopite particles, potentially resulting in more negative zeta potential. However, according to XPS and FTIR results, ligands are present on the surface. Thus, the first mechanism seems more feasible. Further in situ investigations would be required to confirm the mechanism.

Ultrasonic waves can effectively remove impurities from mineral surfaces by inducing cavitation. The pressure and mechanical action from cavitation can disrupt both physical and chemical bonds between the surface and the complexes, facilitating their release. This process can increase the total extent of dissolution by ensuring that more complexes are released from the surface.^37,71^ Thus, after the formation of surface complexes such as >Si–O–cation–citrate, the subsequent release of cation-citrate complexes from the surface may be enhanced by the additional impact of sonication. This process could facilitate the release of the complex, consequently increasing the total extent of dissolution. In addition, it provides the opportunity for the formation of new complexes with the newly cleaned surface. In contrast, in the case of ethylenediamine, there is a direct interaction between the negatively charged silicate surface and the positively charged ethylenediamine (i.e., >Si–O–ethylenediamine), as demonstrated by zeta potential results. This interaction may partly prevent the adsorption of K and Mg on the surface, without significantly increasing the total extent of dissolution and not being strongly influenced by sonication, as observed in the dissolution results.

The findings of this study can be related to other minerals rich in Mg, Si and Fe, such as olivine. The dissolution behavior of phlogopite is influenced by its mineral structure, with elements like K, Fe, Mg, Al, and Si being released to different extents depending on the experimental conditions. Similarly, studies on olivine have demonstrated that its dissolution is also controlled by its mineral composition and structure, with key elements being Mg and Fe.^58^ By comparing the dissolution processes of phlogopite and olivine, further insights can be gained into the mechanisms governing mineral dissolution in the presence of organic ligands such as carboxylates and amines, as well as ultrasonic waves. Future research could explore these comparisons in more detail to validate and expand upon the findings of this study. This approach not only enhances our understanding of mineral dissolution but also opens up potential applications in the cement industry, fertilization, and CO_2_ sequestration, thereby contributing to broader environmental and industrial contexts.

## 5 Conclusions

The enhanced dissolution and extraction percentage of elements from phlogopite increase its utilization potential in different applications in line with circular economy principles. Our findings show that there is great synergy when using a combined sonication and ligand-assisted dissolution process. The importance of pH is evident, as the extraction rates of elements were considerably greater with an initial pH of 4 compared to pH 7. The enhancement due to the combined effect of ultrasound waves and organic ligands varied depending on pH and ligand type, with organic ligands containing carboxylate functional groups being more efficient compared to ethylenediamine. Notably, in the case of S-Cit4, the effect reached as high as 25%. The results suggest that ligands containing carboxylate groups, such as citrate and oxalate, can form surface complexes with cations on the silicate surface. These surface complexes may accelerate dissolution, an effect that is further enhanced by sonication. The dissolution process is affected by the surface composition of phlogopite, the interactions between organic ligands and the surface, and the accumulation of elements on the surface as well. However, further investigations, specifically in situ studies, are required to obtain a deeper understanding of the mechanisms of these phenomena.

## Supplementary Material



## Data Availability

The raw data is available free of charge at [DOI: 10.23729/bb4f4040–8cbc-48ad-9e5e-5e98984b50f3].

## Notes

Supporting Information document provides FTIR spectra and TEM-images of additional samples (PDF)

## §

Department of Chemistry, University of Helsinki, A.I. Virtasenaukio1, 00014 Helsinki, Finland
