# Supplementary material for: Combined Role of Organic Ligands and Ultrasound on the Dissolution of Phlogopite at pH 4 and 7
Source: Langmuir. 2025 Jun 6;41(23):14607–17. doi: 10.1021/acs.langmuir.4c04307 (PMC12177917; doi:10.1021/acs.langmuir.4c04307)
Supplement: Supplementary file 1 [file la4c04307_si_001.pdf]

Supplementary Information

The Combined Role of Organic Ligands and Ultrasound on the Dissolution of Phlogopite at pH 4 and 7

Mahtab Akbarzadeh Khoei[a], Recep Kurtulus[a,b], Mohammad I.M. Alzeer[a,#], Juho Antti Sirviö[a], Juho Yliniemi[a]*

a) Fibre and Particle Engineering Research Unit, University of Oulu, P.O. Box 4300, 90014, Oulu, Finland.

b) Afyon Kocatepe University, Faculty of Engineering, Department of Materials Science and Engineering, Afyonkarahisar, Türkiye.

[#] Current address: Department of Chemistry, University of Helsinki, A.I. Virtasenaukio1, 00014, Helsinki, Finland.

*Email: Juho.yliniemi@oulu.fi

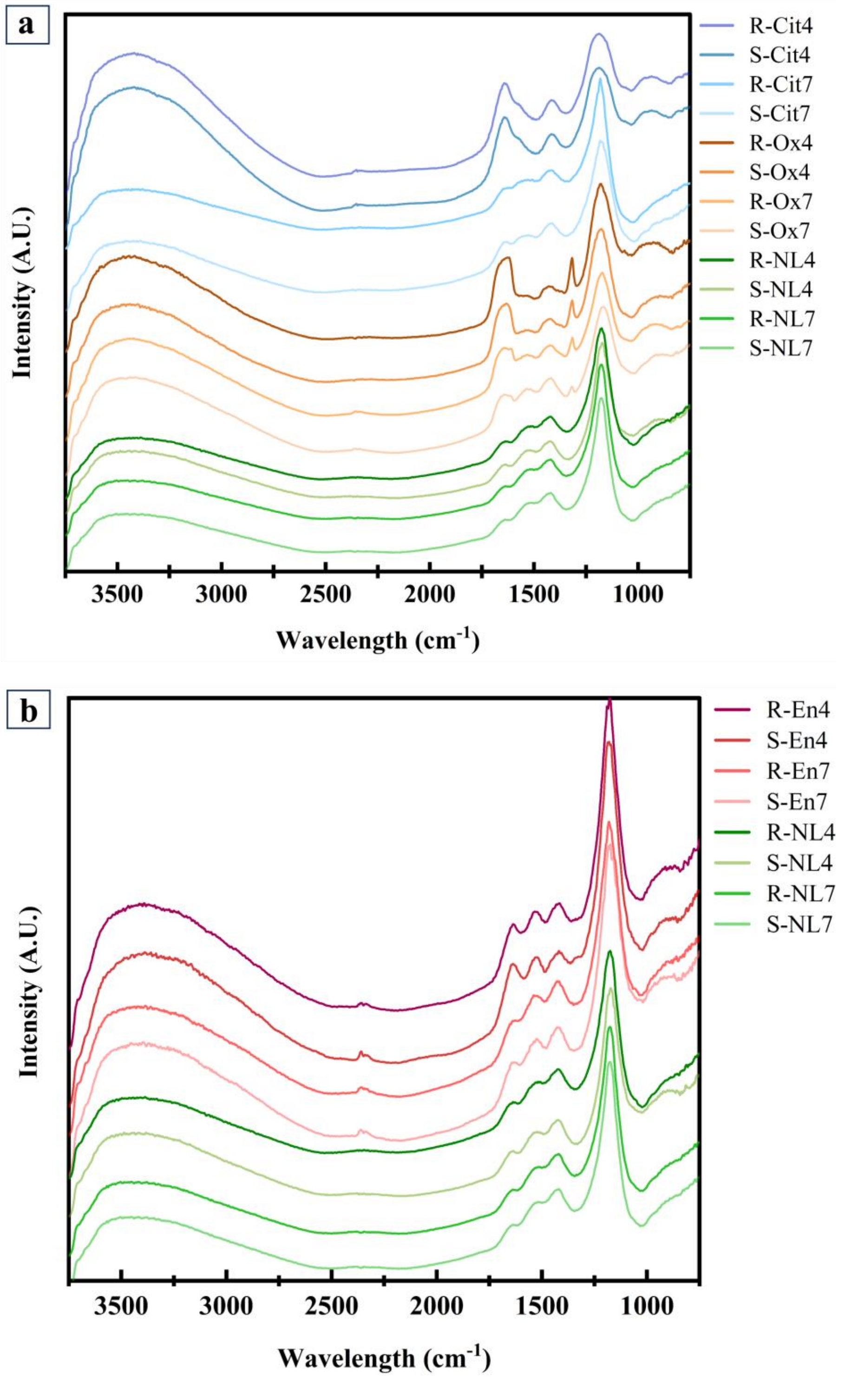

Figure S1. FTIR spectra of a) oxalate, citrate, and reference samples, and b) ethylenediamine and reference samples. The sample codes are abbreviated according to their initial pH (4 or 7), mixing method (stirrer = R or sonicated = S), and the ligand used (NL = no ligand, Cit = citrate, Ox = oxalate, and En = ethylenediamine).

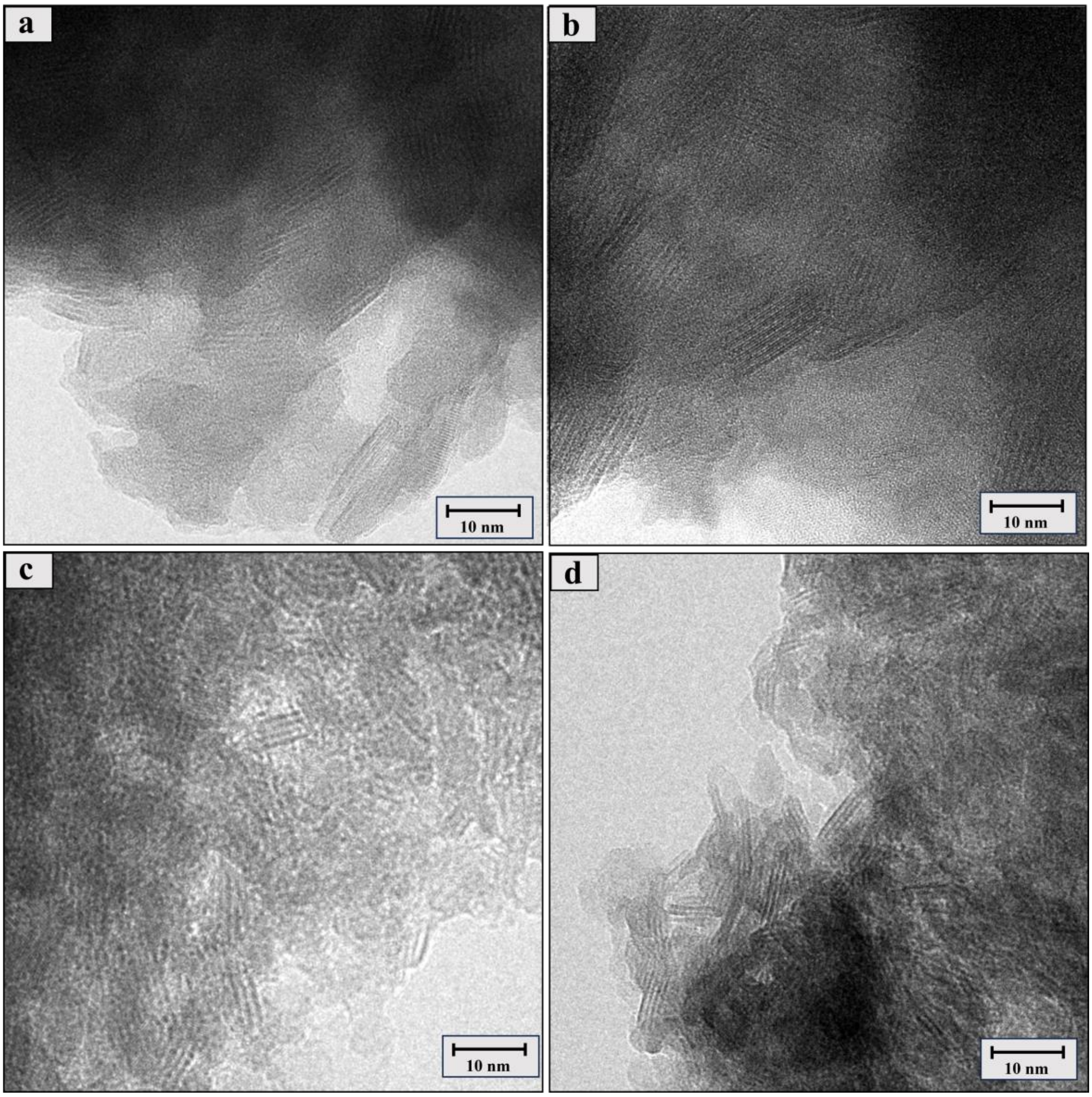

Figure S2. Morphological investigation of samples a) R-Ox4, b) R-EN4, c) S-OX4, and d) S-EN4. The sample codes are abbreviated according to their initial pH (4 or 7), mixing method (stirrer = R or sonicated = S), and the ligand used (Ox = oxalate, and En = ethylenediamine).
